# Supplementary material for: Gestational weight gain and its effect on birth outcomes in sub-Saharan Africa: Systematic review and meta-analysis
Source: PLoS One. 2020 Apr 23;15(4):e0231889. doi: 10.1371/journal.pone.0231889 (PMC7179909; doi:10.1371/journal.pone.0231889)
Supplement: S1 Table — (DOCX) [file pone.0231889.s001.docx]

S1 Table: PRISMA-P 2015 checklist: recommended items to include in a systematic review: recommended items to address in a systematic review protocol*.

| **Section/topic** | **Item #** | **Checklist item** | Self-Evaluation |
| --- | --- | --- | --- |
| **ADMINISTRATIVE INFORMATION** | | |  |
| **Title** | | |  |
| **Identification** | 1a | Identify the report as a protocol of a systematic review | YES, identified ( page 1) |
| **Update** | 1b | If the protocol is for an update of a previous systematic review, identify as such | Not applicable |
| **Registration** | 2 | If registered, provide the name of the registry (e.g., PROSPERO) and registration number | PROSPERO “CRD42018085499” (page 4) |
| **Authors** | | |  |
| **Contact** | 3a | Provide name, institutional affiliation, and e-mail address of all protocol authors; provide physical mailing address of corresponding author | YES, it was provided (page 1) |
| **Contributions** | 3b | Describe contributions of protocol authors and identify the guarantor of the review | YES, this was provided (page27) |
| **Amendments** | 4 | If the protocol represents an amendment of a previously completed or published protocol, identify as such and list changes; otherwise, state plan for documenting important protocol amendments | Not applicable |
| **Support** | | |  |
| **Sources** | 5a | Indicate sources of financial or other support for the review | Not applicable |
| **Sponsor** | 5b | Provide name for the review funder and/or sponsor | Not applicable |
| **Role of sponsor/funder** | 5c | Describe roles of funder(s), sponsor(s), and/or institution(s), if any, in developing the protocol | Not applicable |
| **INTRODUCTION** | | |  |
| **Rationale** | 6 | Describe the rationale for the review in the context of what is already known | Yes, this was done (page 3) |
| **Objectives** | 7 | Provide an explicit statement of the question(s) the review will address with reference to participants, interventions, comparators, and outcomes (PICO) | Yes this was clearly stated (page 5) |
| **METHODS** | | |  |
| **Eligibility criteria** | 8 | Specify the study characteristics (e.g., PICO, study design, setting, time frame) and report characteristics (e.g., years considered, language, publication status) to be used as criteria for eligibility for the review | Yes, this was done (page 4) |
| **Information sources** | 9 | Describe all intended information sources (e.g., electronic databases, contact with study authors, trial registers, or other grey literature sources) with planned dates of coverage | Yes this was well described (page 4) |
| **Search strategy** | 10 | Present draft of search strategy to be used for at least one electronic database, including planned limits, such that it could be repeated | Yes this provided as additional file |
| **Study records** | | |  |
| **Data management** | 11a | Describe the mechanism(s) that will be used to manage records and data throughout the review | Yes this was described |
| **Selection process** | 11b | State the process that will be used for selecting studies (e.g., two independent reviewers) through each phase of the review (i.e., screening, eligibility, and inclusion in meta-analysis) | Yes this was stated  (page 4, 6 and 8) |
| **Data collection process** | 11c | Describe planned method of extracting data from reports (e.g., piloting forms, done independently, in duplicate), any processes for obtaining and confirming data from investigators | Yes this was described  (page 4, 6 and 8) |
| **Data items** | 12 | List and define all variables for which data will be sought (e.g., PICO items, funding sources), any pre-planned data assumptions and simplifications | Yes this was provided  ( page 5) |
| **Outcomes and prioritization** | 13 | List and define all outcomes for which data will be sought, including prioritization of main and additional outcomes, with rationale | Yes this was done  ( page 5) |
| **Risk of bias in individual studies** | 14 | Describe anticipated methods for assessing risk of bias of individual studies, including whether this will be done at the outcome or study level, or both; state how this information will be used in data synthesis | Yes this was done (page 9) |
| **Data** | | |  |
| **Synthesis** | 15a | Describe criteria under which study data will be quantitatively synthesized | Yes this was described (Page 7) |
|  | 15b | If data are appropriate for quantitative synthesis, describe planned summary measures, methods of handling data, and methods of combining data from studies, including any planned exploration of consistency (e.g., *I*^2^, Kendall’s tau) | Yes this was stated (Page 7) |
|  | 15c | Describe any proposed additional analyses (e.g., sensitivity or subgroup analyses, meta-regression) | Yes this was described (pages 7 and 26) |
|  | 15d | If quantitative synthesis is not appropriate, describe the type of summary planned | Yes this was described (Page 7) |
| **Meta-bias(es)** | 16 | Specify any planned assessment of meta-bias(es) (e.g., publication bias across studies, selective reporting within studies) | Yes this was described |
| **Confidence in cumulative evidence** | 17 | Describe how the strength of the body of evidence will be assessed (e.g., GRADE) | Yes (EPHPP) (pages 5 and Table 2) |

*** It is strongly recommended that this checklist be read in conjunction with the PRISMA-P Explanation and Elaboration (cite when available) for important clarification on the items. Amendments to a review protocol should be tracked and dated. The copyright for PRISMA-P (including checklist) is held by the PRISMA-P Group and is distributed under a Creative Commons Attribution Licence 4.0.**

*From: Shamseer L, Moher D, Clarke M, Ghersi D, Liberati A, Petticrew M, Shekelle P, Stewart L, PRISMA-P Group. Preferred reporting items for systematic review and meta-analysis protocols (PRISMA-P) 2015: elaboration and explanation. BMJ. 2015 Jan 2;349 (jan02 1):g7647.*
