# Supplementary material for: Gestational weight gain and its effect on birth outcomes in sub-Saharan Africa: Systematic review and meta-analysis
Source: PLoS One. 2020 Apr 23;15(4):e0231889. doi: 10.1371/journal.pone.0231889 (PMC7179909; doi:10.1371/journal.pone.0231889)
Supplement: S2 Table — (DOCX) [file pone.0231889.s002.docx]

Table 2: Search strategies with corresponding database and numbers of articles accessed

|  | Database | Combined search phrase | Search results |
| --- | --- | --- | --- |
| 1 | PubMed  (date 16/12/2019) | ((((((((((pregnan*[Title/Abstract]) OR Gestation*)) AND ((weight gain[Title/Abstract]) OR Weight change[Title/Abstract])) AND (((((((((((((((((((((((((((((((((((((((((((((((((((((Sub-Saharan[Title/Abstract]) OR Subsaharan Africa[Title/Abstract]) OR Low income country[Title/Abstract]) OR Developing country[Title/Abstract]) OR Angola[Title/Abstract]) OR Benin[Title/Abstract]) OR Botswana[Title/Abstract]) OR Burkina Faso[Title/Abstract]) OR Burundi[Title/Abstract]) OR Cameroon[Title/Abstract]) OR Cape Verde[Title/Abstract]) OR Central African Republic[Title/Abstract]) OR Chad[Title/Abstract]) OR Comoros[Title/Abstract]) OR Congo[Title/Abstract]) OR Côte d'Ivoire[Title/Abstract]) OR Djibouti[Title/Abstract]) OR Equatorial Guinea[Title/Abstract]) OR Eritrea[Title/Abstract]) OR Ethiopia[Title/Abstract]) OR Gabon[Title/Abstract]) OR Gambia[Title/Abstract]) OR Ghana[Title/Abstract]) OR Guinea[Title/Abstract]) OR Guinea-Bissau[Title/Abstract]) OR Kenya[Title/Abstract]) OR Lesotho[Title/Abstract]) OR Liberia[Title/Abstract]) OR Madagascar[Title/Abstract]) OR Malawi[Title/Abstract]) OR Mali[Title/Abstract]) OR Mauritania[Title/Abstract]) OR Mauritius[Title/Abstract]) OR Mozambique[Title/Abstract]) OR Namibia[Title/Abstract]) OR Niger[Title/Abstract]) OR Nigeria[Title/Abstract]) OR Réunion[Title/Abstract]) OR Rwanda[Title/Abstract]) OR (Sao Tome[Title/Abstract] AND Principe[Title/Abstract])) OR Senegal[Title/Abstract]) OR Seychelles[Title/Abstract]) OR Sierra Leone[Title/Abstract]) OR Somalia[Title/Abstract]) OR South Africa[Title/Abstract]) OR Sudan[Title/Abstract]) OR Swaziland[Title/Abstract]) OR Tanzania[Title/Abstract]) OR Togo[Title/Abstract]) OR Uganda[Title/Abstract]) OR Western Sahara[Title/Abstract]) OR Zambia[Title/Abstract]) OR Zimbabwe[Title/Abstract]))) AND ( "1990/01/01"[PDat] : "2019/12/31"[PDat] ))) AND ( "1990/01/01"[PDat] : "2019/12/31"[PDat] ) AND Humans[Mesh]) Filters: Publication date from 1990/01/01 to 2019/12/31; Humans; English | 121 |
| 2 | Scopus  (date 12/12/19) | ( ( "Gestation*" OR "Pregnancy" ) AND ( "weight gain" ) ) AND ( "angola" OR "benin" OR "botswana" OR "Burkina faso" OR "cameroon" OR "cape verde" OR "central African republic" OR "chad" OR "comoros" AND "congo" OR "cote d'ivoire" OR "djibouti" OR "equatorial guinea" OR "eritrea" OR "ethiopia" OR "gabon" OR "gambia" OR "ghana" OR "guinea" OR "guineabissau" OR "kenya" OR "lesotho" OR "liberia" OR "madagascar" OR "malawi"OR "mali" OR "mauritania" OR "mauritius" OR "mozambique" OR "namibia" OR "niger" OR "nigeria" OR "réunion" OR "rwanda" OR "sao tome and principe" OR "senegal" OR "seychelles" OR "sierra leone" OR "somalia" OR "south africa" OR "sudan" OR "swaziland" OR "tanzania" OR "togo" OR "uganda" OR "western sahara" OR "zambia" OR "zimbabwe" OR "Sub Saharan Afrrica" OR "Developing countries" )  AND (LIMIT TO ( PUBYEAR , 2019 ) OR LIMIT TO ( PUBYEAR , 2018 ) OR LIMIT- TO ( PUBYEAR , 2017 ) OR LIMIT TO ( PUBYEAR , 2016 ) OR LIMIT-TO ( PUBYEAR , 2015 ) OR LIMIT TO ( PUBYEAR , 2014 ) OR LIMIT-TO ( PUBYEAR , 2013 ) OR LIMIT TO ( PUBYEAR , 2012 ) OR LIMIT-TO ( PUBYEAR , 2011 ) OR LIMIT-TO ( PUBYEAR , 2010 ) OR LIMIT-TO ( PUBYEAR , 2009 ) OR LIMIT-TO ( PUBYEAR , 2008 ) OR LIMIT-TO ( PUBYEAR , 2007 ) OR LIMIT-TO ( PUBYEAR , 2006 ) OR LIMIT-TO ( PUBYEAR , 2005 ) OR LIMIT-TO ( PUBYEAR , 2004 ) OR LIMIT-TO ( PUBYEAR , 2003 ) OR LIMIT-TO ( PUBYEAR , 2002 ) OR LIMIT-TO ( PUBYEAR , 2001 ) OR LIMIT-TO ( PUBYEAR , 2000 ) OR LIMIT-TO ( PUBYEAR , 1999 ) OR LIMIT-TO ( PUBYEAR , 1998 ) OR LIMIT-TO ( PUBYEAR , 1997 ) OR LIMIT-TO ( PUBYEAR , 1996 ) OR LIMIT-TO ( PUBYEAR , 1995 ) OR LIMIT-TO ( PUBYEAR , 1993 ) OR LIMIT-TO ( PUBYEAR , 1992 ) OR LIMIT-TO ( PUBYEAR , 1991 ) OR LIMIT-TO ( PUBYEAR , 1990 ) ) AND ( LIMIT-TO ( LANGUAGE , "English" ) ) AND (EXCLUDE (DOCTYPE “re”) OR EXCLUDE (DOCTYPE “bk”) OR EXCLUDE (DOCTYPE “ch”) OR EXCLUDE (DOCTYPE “no”) OR EXCLUDE (DOCTYPE “sh”)) | 401 |
|  |  |  |  |
| 3 | Medline (Ovid) | \| # \| Searches (date 15/12/2019) \| Results \| \| --- \| --- \| --- \| \| 1 \| exp Pregnancy/ or Gestation*.mp. \| 953412 \| \| 2 \| weight gain.mp. or exp Weight Gain/ \| 72455 \| \| 3 \| 1 and 2 \| 11743 \| \| 4 \| Angola.mp. or exp Angola/ \| 1543 \| \| 5 \| Benin.mp. or exp Benin/ \| 3719 \| \| 6 \| Botswana.mp. or exp Botswana/ \| 2527 \| \| 7 \| Burkina Faso.mp. or exp Burkina Faso/ \| 4356 \| \| 8 \| Burundi.mp. or exp Burundi/ \| 947 \| \| 9 \| Cameroon.mp. or exp Cameroon/ \| 7540 \| \| 10 \| Cape Verde.mp. or exp Cape Verde/ \| 551 \| \| 11 \| Central African Republic.mp. or exp Central African Republic/ \| 1176 \| \| 12 \| Chad.mp. or exp Chad/ \| 1260 \| \| 13 \| Comoros.mp. or exp Comoros/ \| 485 \| \| 14 \| Congo.mp. or exp Congo/ or exp "Democratic Republic of the Congo"/ \| 14587 \| \| 15 \| Cote d'Ivoire.mp. or exp Cote d'Ivoire/ \| 3798 \| \| 16 \| Djibouti.mp. or exp Djibouti/ \| 404 \| \| 17 \| Equatorial Guinea.mp. or exp Equatorial Guinea/ \| 456 \| \| 18 \| exp Africa/ or exp Eritrea/ or exp Ethiopia/ or Eritrea .mp. or exp Developing Countries/ \| 312978 \| \| 19 \| Gabon.mp. or exp Gabon/ \| 2041 \| \| 20 \| Gambia.mp. or exp Gambia/ \| 3095 \| \| 21 \| Ghana.mp. or exp Ghana/ \| 10972 \| \| 22 \| Guinea.mp. or exp Guinea-Bissau/ or exp Guinea/ or exp Equatorial Guinea/ or exp Papua New Guinea/ or exp New Guinea/ \| 161589 \| \| 23 \| Kenya.mp. or exp Kenya/ \| 20693 \| \| 24 \| Lesotho.mp. or exp Lesotho/ \| 712 \| \| 25 \| Liberia.mp. or exp Liberia/ \| 1844 \| \| 26 \| Madagascar.mp. or exp Madagascar/ \| 5130 \| \| 27 \| Malawi.mp. or exp Malawi/ \| 7187 \| \| 28 \| Mali.mp. or exp Mali/ \| 3895 \| \| 29 \| Mauritania.mp. or exp Mauritania/ \| 692 \| \| 30 \| Mauritius.mp. or exp Mauritius/ \| 1039 \| \| 31 \| Mozambique.mp. or exp Mozambique/ \| 3660 \| \| 32 \| Namibia.mp. or exp Namibia/ \| 1691 \| \| 33 \| exp Niger/ or Niger.mp. \| 13201 \| \| 34 \| Nigeria.mp. or exp Nigeria/ \| 35607 \| \| 35 \| Reunion.mp. or Reunion/ \| 3262 \| \| 36 \| Rwanda.mp. or exp Rwanda/ \| 3275 \| \| 37 \| Senegal.mp. or exp Senegal/ \| 7493 \| \| 38 \| Seychelles.mp. or exp Seychelles/ \| 718 \| \| 39 \| Sierra Leone.mp. or exp Sierra Leone/ \| 2346 \| \| 40 \| Somalia.mp. or exp Somalia/ \| 2140 \| \| 41 \| South Africa.mp. or exp South Africa/ \| 51239 \| \| 42 \| exp Sudan/ or exp South Sudan/ or Sudan.mp. \| 9060 \| \| 43 \| Swaziland.mp. or exp Swaziland/ \| 894 \| \| 44 \| Tanzania.mp. or exp Tanzania/ \| 14346 \| \| 45 \| Togo.mp. or exp Togo/ \| 1654 \| \| 46 \| Uganda.mp. or exp Uganda/ \| 15646 \| \| 47 \| Western Sahara.mp. \| 62 \| \| 48 \| Zambia.mp. or exp Zambia/ \| 5947 \| \| 49 \| Zimbabwe.mp. or exp Zimbabwe/ \| 7396 \| \| 50 \| 4 or 5 or 6 or 7 or 8 or 9 or 10 or 11 or 12 or 13 or 14 or 15 or 16 or 17 or 18 or 19 or 20 or 21 or 22 or 23 or 24 or 25 or 26 or 27 or 28 or 29 or 30 or 31 or 32 or 33 or 34 or 35 or 36 or 37 or 38 or 39 or 40 or 41 or 42 or 43 or 44 or 45 or 46 or 47 or 48 or 49 \| 539283 \| \| 51 \| 3 and 50 \| 325 \| \| 52 \| Limit 51 to (english language and yr=“1990-2019”) \| 254 \| | 254 |
|  |  | Searches (15/12/2019) Results |  |
|  | Embase  (Excerpta medica) | \| # \|  \|  \| \| --- \| --- \| --- \| \| 1 \| gestational OR pregnancy \| 1037996 \| \| 2 \| weight AND gain \| 143224 \| \| 3 \| 'africa south of the sahara' \| 13664 \| \| 4 \| 'africa' \| 307658 \| \| 5 \| 'developing country' \| 96957 \| \| 6 \| 'sub-Saharan' \| 28917 \| \| 7 \| 'angola' \| 2230 \| \| 8 \| 'benin' \| 11982 \| \| 9 \| 'botswana' \| 4482 \| \| 10 \| 'burkina faso' \| 7609 \| \| 11 \| 'burundi' \| 1246 \| \| 12 \| 'cameroon' \| 14134 \| \| 13 \| 'cape verde' \| 675 \| \| 14 \| 'central african republic' \| 1674 \| \| 15 \| 'chad' \| 19859 \| \| 16 \| 'comoros' \| 534 \| \| 17 \| 'congo' \| 21555 \| \| 18 \| 'ivory cost' \| 27 \| \| 19 \| djibouti \| 604 \| \| 20 \| 'equatorial guinea' \| 702 \| \| 21 \| 'eritrea' \| 3576 \| \| 22 \| 'ethiopia' \| 23108 \| \| 23 \| 'gabon' \| 3648 \| \| 24 \| 'gambia' \| 5116 \| \| 25 \| 'ghana' \| 19338 \| \| 26 \| 'kenya' \| 50276 \| \| 27 \| 'lesotho' \| 1040 \| \| 28 \| 'liberia' \| 2575 \| \| 29 \| 'madagascar' \| 6894 \| \| 30 \| 'malawi' \| 11131 \| \| 31 \| 'mali' \| 9306 \| \| 32 \| 'mauritania' \| 959 \| \| 33 \| 'mauritius' \| 2325 \| \| 34 \| 'mozambique' \| 5748 \| \| 35 \| 'namibia' \| 2756 \| \| 36 \| 'niger' \| 28404 \| \| 37 \| 'nigeria' \| 89631 \| \| 38 \| 'rwanda' \| 5125 \| \| 39 \| 'senegal' \| 15802 \| \| 40 \| 'seychelles' \| 974 \| \| 41 \| 'sierra leone' \| 3196 \| \| 42 \| 'somalia' \| 2529 \| \| 43 \| 'south africa' \| 192120 \| \| 44 \| 'sudan' \| 15233 \| \| 45 \| 'swaziland' \| 1839 \| \| 46 \| 'tanzania' \| 21679 \| \| 47 \| 'togo' \| 4507 \| \| 48 \| 'uganda' \| 28921 \| \| 49 \| 'western sahara' \| 91 \| \| 50 \| 'zambia' \| 9518 \| \| 51 \| 'zimbabwe' \| 13251 \| \| 52 \| #1 AND #2 \| 13048 \| \| 53 \| #3 OR #4 OR #5 OR #6 OR #7 OR #8 OR #9 OR #10 OR #11 OR #12 OR #13 OR #14 OR #15 OR #16 OR #17 OR #18 OR #19 OR #20 OR #21 OR #22 OR #23 OR #24 OR #25 OR #26 OR #27 OR #28 OR #29 OR #30 OR #31 OR #32 OR #33 OR #34 OR #35 OR #36 OR #37 OR #38 OR #39 OR #40 OR #41 OR #42 OR #43 OR #44 OR #45 OR #46 OR #47 OR #48 OR #49 OR #50 OR #51 \| 692514 \| \| 54 \| #52 AND #53 AND [english]/lim AND [1990-2019]/py \| 584 \| | 584 |
|  |  |  |  |

| 5 | CINAHL (Date 16/12/2019) | Limiters/Expanders | Last Run Via | Results |
| --- | --- | --- | --- | --- |
| S8 | S1 AND S7 | **Limiters** - Published Date: 19900101-20191231; Language: English  **Expanders** - Apply related words; Also search within the full text of the articles; Apply equivalent subjects  **Search modes** - Boolean/Phrase | Interface - EBSCOhost Research Databases  Search Screen - Advanced Search  Database - CINAHL | 36 |
| S7 | S2 OR S3 OR S4 OR S5 OR S6 | **Expanders** - Apply related words; Also search within the full text of the articles; Apply equivalent subjects  **Search modes** - Boolean/Phrase | Interface - EBSCOhost Research Databases  Search Screen - Advanced Search  Database - CINAHL | 47,065 |
| S6 | AB Zambia OR AB Zimbabwe OR AB sub-Saharan Africa OR AB Developing Country | **Expanders** - Apply related words; Also search within the full text of the articles; Apply equivalent subjects  **Search modes** - Boolean/Phrase | Interface - EBSCOhost Research Databases  Search Screen - Advanced Search  Database - CINAHL | 17,262 |
| S5 | AB Senegal OR AB Seychelles OR AB Sierra Leone OR AB Somalia OR AB South Africa OR AB Sudan OR AB South Sudan OR AB Swaziland OR AB Tanzania OR AB Togo OR AB Uganda OR AB Western Sahara | **Expanders** - Apply related words; Also search within the full text of the articles; Apply equivalent subjects  **Search modes** - Boolean/Phrase | Interface - EBSCOhost Research Databases  Search Screen - Advanced Search  Database - CINAHL | 14,940 |
| S4 | AB Liberia OR AB Madagascar OR AB Malawi OR AB Mali OR AB Mauritania OR AB Mauritius OR AB Mozambique OR AB Namibia OR AB Niger OR AB NIgeria OR AB Rwanda | **Expanders** - Apply related words; Also search within the full text of the articles; Apply equivalent subjects  **Search modes** - Boolean/Phrase | Interface - EBSCOhost Research Databases  Search Screen - Advanced Search  Database - CINAHL | 8,637 |
| S3 | AB Djibouti OR AB Equatorial Guinea OR AB Eritrea OR AB Ethiopia OR AB Gabon OR AB Gambia OR AB Ghana OR AB Guinea OR AB Guinea-Bissau OR AB Equatorial Guinea OR AB Kenya OR AB Lesotho | **Expanders** - Apply related words; Also search within the full text of the articles; Apply equivalent subjects  **Search modes** - Boolean/Phrase | Interface - EBSCOhost Research Databases  Search Screen - Advanced Search  Database - CINAHL | 10,567 |
| S2 | AB Angola OR AB Benin OR AB Botswana OR AB Burkina Faso OR AB Burundi OR AB Cameroon OR AB Cape Verde OR AB Central African Republic OR AB Chad OR AB Comoros OR AB Democratic Republic of the Congo OR AB Cote d'Ivoire | **Expanders** - Apply related words; Also search within the full text of the articles; Apply equivalent subjects  **Search modes** - Boolean/Phrase | Interface - EBSCOhost Research Databases  Search Screen - Advanced Search  Database - CINAHL | 3479 |
| S1 | AB Gestational weight gain OR AB pregnancy weight gain OR AB weight gain during pregnancy | **Expanders** - Apply related words; Also search within the full text of the articles; Apply equivalent subjects  **Search modes** - Boolean/Phrase | **Interface** - EBSCOhost Research Databases **Search Screen** - Advanced Search **Database** - CINAHL | 2,014 |
