# Supplementary material for: Gestational weight gain and its effect on birth outcomes in sub-Saharan Africa: Systematic review and meta-analysis
Source: PLoS One. 2020 Apr 23;15(4):e0231889. doi: 10.1371/journal.pone.0231889 (PMC7179909; doi:10.1371/journal.pone.0231889)
Supplement: S3 Table — (DOCX) [file pone.0231889.s003.docx]

S3 Table: summary result of meta-analyses (Effect of inadequate GWG on Birth outcome)

| S.N | Type of Outcome | No. Studies | Total number of Inadequate GWG | Inadequate GWG with outcome | Total number of adequate GWG | adequate GWG with outcome | RR 95% CI | *I^2^* |
| --- | --- | --- | --- | --- | --- | --- | --- | --- |
|  | Caesarean section | 2 | 315 | 56 | 245 | 88 | 0.58 [0.21, 1.68] | 90% |
|  | Obstetric haemorrhage | 2 | 315 | 21 | 345 | 21 | 1.10[0.19, 6.29] | 77% |
|  | Pre-eclampsia | 2 | 316 | 18 | 241 | 21 | 0.62[0.34, 1.13] | 41% |
|  | Macrosomia | 2 | 315 | 11 | 245 | 17 | 0.50[0.24,1.04] | 0% |
|  | Low birth weight | 2 | 471 | 66 | 246 | 17 | 2.45[0.15, 45.33] | 93% |
