# Supplementary material for: Gestational weight gain and its effect on birth outcomes in sub-Saharan Africa: Systematic review and meta-analysis
Source: PLoS One. 2020 Apr 23;15(4):e0231889. doi: 10.1371/journal.pone.0231889 (PMC7179909; doi:10.1371/journal.pone.0231889)
Supplement: S4 Table — (DOCX) [file pone.0231889.s004.docx]

S4 Table: summary result of meta-analyses (Effect of excessive GWG on Birth outcome)

| S.N | Type of Outcome | No. Studies | Total number of excess GWG | Excess GWG with outcome | Total number of adequate GWG | adequate GWG with outcome | RR 95% CI | *I^2^* |
| --- | --- | --- | --- | --- | --- | --- | --- | --- |
|  | Caesarean section | 2 | 252 | 93 | 245 | 88 | 0.9[0.79, 1.21] | 0% |
|  | Obstetric haemorrhage | 2 | 315 | 21 | 245 | 21 | 1.10[0.19, 6.29] | 77% |
|  | Pre-eclampsia | 2 | 252 | 34 | 246 | 21 | 2.47[0.24, 25.78] | 89% |
|  | Macrosomia | 2 | 252 | 26 | 245 | 17 | 1.47[0.82, 2.63] | 0% |
|  | Low birth weight | 2 | 156 | 10 | 246 | 17 | 0.64[0.31, 1.34] | 0% |
