# Supplementary figures and images for: Gestational weight gain and its effect on birth outcomes in sub-Saharan Africa: Systematic review and meta-analysis
Source: PLoS One. 2020 Apr 23;15(4):e0231889. doi: 10.1371/journal.pone.0231889 (PMC7179909; doi:10.1371/journal.pone.0231889)

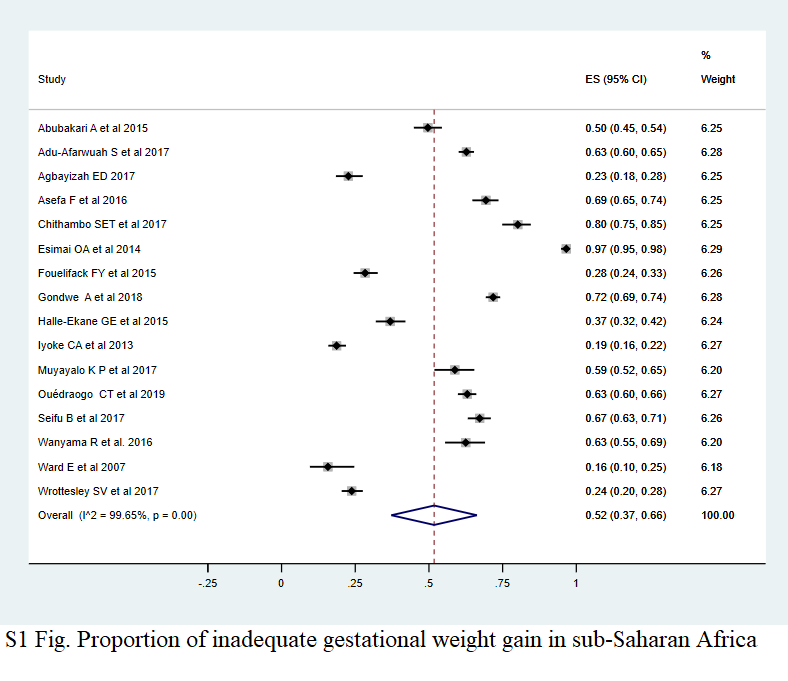

Supplement: S1 Fig — (TIF) [file pone.0231889.s005.tif]

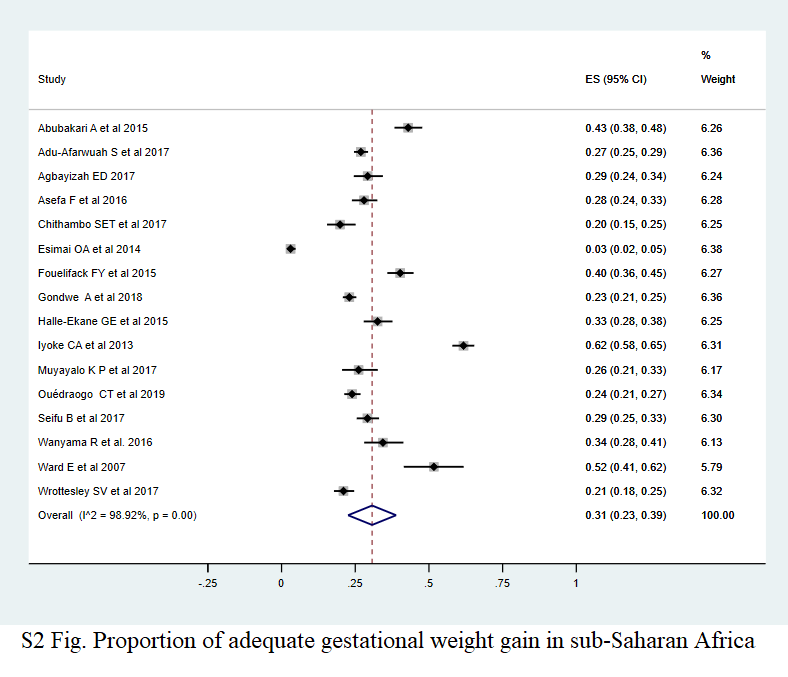

Supplement: S2 Fig — (TIF) [file pone.0231889.s006.tif]

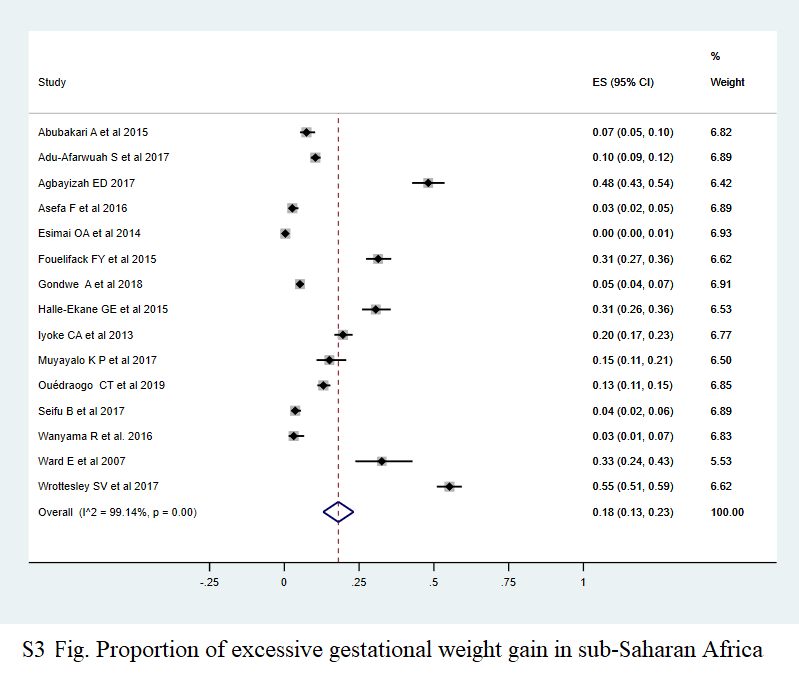

Supplement: S3 Fig — (TIF) [file pone.0231889.s007.tif]

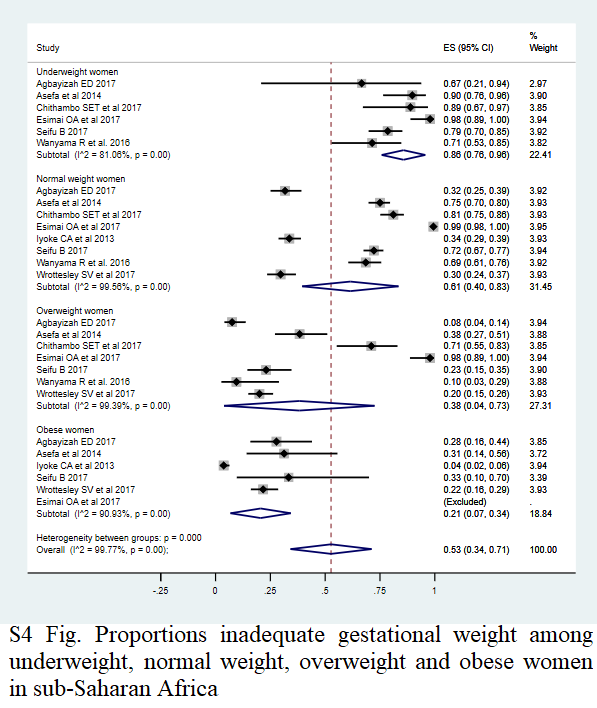

Supplement: S4 Fig — (TIF) [file pone.0231889.s008.tif]

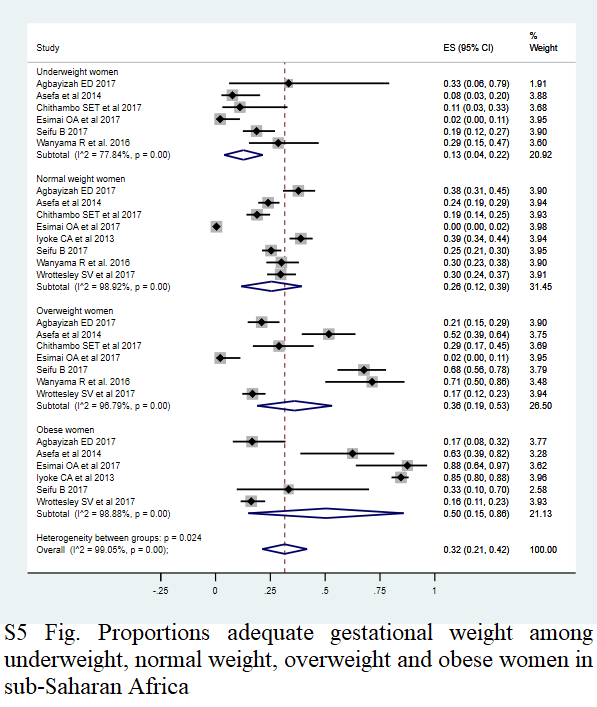

Supplement: S5 Fig — (TIF) [file pone.0231889.s009.tif]

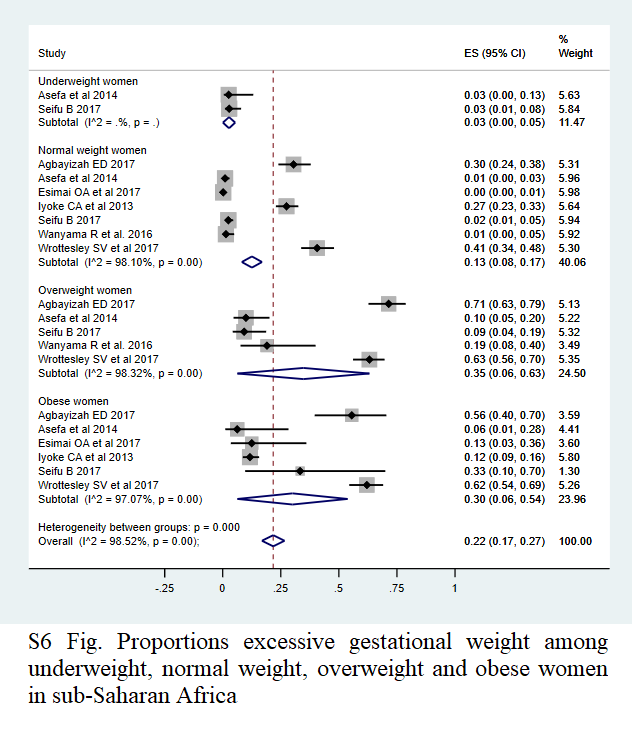

Supplement: S6 Fig — (TIF) [file pone.0231889.s010.tif]
