# Supplementary material for: Gestational weight gain and its effect on birth outcomes in sub-Saharan Africa: Systematic review and meta-analysis
Source: PLoS One. 2020 Apr 23;15(4):e0231889. doi: 10.1371/journal.pone.0231889 (PMC7179909; doi:10.1371/journal.pone.0231889)
Supplement: Attachment — Submitted filename: Comments to authors.pdf [file pone.0231889.s011.pdf]

I reviewed the manuscript “Gestational Weight Gain and its Effect on Birth Outcomes in sub-Saharan Africa: Systematic Review and Meta-analysis”. The author(s) of this paper sought to present a review that determines the distribution of gestational weight gain (GWG) across African countries and its association with birth outcomes. Overall, I think this paper is well-written, and merits consideration for publication.

Some of the papers main strengths include a very clear presentation of findings of the studies considered in the review as well as meticulously critiquing each of the papers, identifying their merits and demerits. I, however, have some minor comments about issues the authors can clarify or address. Some of my concerns are outlined as below:

Minor comments:

Study selection procedure

I strongly suggest that the authors remove the names from the body of the article. Stating that “the authors screened the studies …” should suffice. The authors have clearly spelled out how they each contributed to the article at the end of it. Additionally, I don’t really think disagreements between authors and how they were resolved have any essence in the article. Please remove it.

Data extraction process

Again, remove the names of specific authors. I think all the authors bear responsibility for the merits and the demerits of the methods and procedures used in this study.

Study characteristics

I suggest the authors start a new sentence after Congo before proceeding to present the categories of the countries based on the income status. As it looks now, it reads as if the countries categorized based on income status are in addition to those listed by names. I would suggest starting the new sentence with “Based on the income status, these countries can be grouped into …”

Effect of GWG on birth outcomes

Low birthweight

While the results of Nenomsa et al. [39] show quite clear relationship between GWG and birth weight, Fouelifack et al. [41] seem to present quite a different picture. Were there any reasons presented in the study that could have accounted for a greater proportion of women who gained adequate GWG giving birth to LBW babies than those who did not gain adequate GWG?

Discussion

On page 23, change this sentence in the second paragraph:

"Firstly, the measurement of pre-pregnancy weight of the women was problematic, with four studies [9, 41, 52, 54] using self-reported pre-pregnancy weight~~. However,~~ because there is a typically a difference between self-reported weight and actual measured weight [82-84]."

"Women tend to under-report their pre-pregnancy weight [85, 86], and…"

Substantively, I would suggest caution with attributing studies [85] and [86] to a study in sub-Saharan Africa. This is because I believe there is a social and cultural component that may render this statement not necessarily true for African women (there is quite a lot I could try to explain here but I will let you do that work on your end). Weight gain is not necessarily perceived as negative as it is in countries like the USA. I, therefore, suspect that women will be less likely inclined to deliberate understate their weight.

Summing it all up

I enjoyed reading the paper. It critiques that other studies very well, while it also does a significant amount of work outlining its own limitations. I suggest you consider the comments above and it will make an interesting read for your audience.
