# Supplementary material for: Gestational weight gain and its effect on birth outcomes in sub-Saharan Africa: Systematic review and meta-analysis
Source: PLoS One. 2020 Apr 23;15(4):e0231889. doi: 10.1371/journal.pone.0231889 (PMC7179909; doi:10.1371/journal.pone.0231889)
Supplement: Attachment — Submitted filename: Response to Reviewers.docx [file pone.0231889.s012.docx]

Title:	Gestational Weight Gain and its Effect on Birth Outcomes in sub-Saharan Africa: Systematic Review and Meta-analysis

Authors

1. Fekede Asefa Kumsa: [sinboona@gmail.com](mailto:sinboona@gmail.com)
2. Allison Cummins: [Allison.cummins@uts.edu.au](mailto:Allison.cummins@uts.edu.au)
3. Yadeta Dessie: [yad_de2005@yahoo.com](mailto:yad_de2005@yahoo.com)
4. Andrew Hayen: [Andrew.hayen@uts.edu.au](mailto:Andrew.hayen@uts.edu.au)
5. Maralyn Foureur: [Maralyn.Foureur@health.nsw.gov.au](mailto:Maralyn.Foureur@health.nsw.gov.au)

Dear editor,

We are very grateful to the editor and all the peer reviewers who provided crucial comments and feedback on the manuscript which has helped us to improve and enrich the manuscript. We have revised the manuscript based on the comments and we have provided a point-by-point description of the amendments made on the manuscript in the following table.

Sincerely

| **Journal requirements** | **Authors' reply** |
| --- | --- |
| 1. Please ensure that your manuscript meets PLOS ONE's style requirements, including those for file naming | We have edited the manuscript to adhere to PloS One's style requirements. |
| 2. Please ensure that your search strategy includes studies that have been published in the past 12 months. If this is not appropriate please justify the reasons within the text. | We have updated our search and included five more papers that were been published in the last two years. |
| 3. Please include captions for your Supporting Information files at the end of your manuscript, and update any in-text citations to match accordingly | Thank you. We have put supporting information at the end of the manuscript (on page 38) and updated in-text citations |
| 4. Please use the space provided to explain your answers to the questions above. You may also include additional comments for the author, including concerns about dual publication, research ethics, or publication ethics | We have explained each point raised by reviewers in the following tables. We found one dual publication. Wanyama R, Obai G, Odongo P, Kagawa MN, Baingana RK. Are Women In Low Income Setting Gaining Adequate Gestational Weight? A Prospective Cross Sectional Study In Urban Uganda. Journal of Public Health. 2016; 2 (11):1-14; and Wanyama R, Obai G, Odongo P, Kagawa MN, Baingana RK. Are women in Uganda gaining adequate gestational weight? A prospective study in low income urban Kampala. Reproductive health. 2018; 15 (1):160’. We excluded the second paper because we had already extracted the data from the former publication. |

| **Reviewer 1 comments** | **Authors' reply** |
| --- | --- |
| 1. Follow the Socioecological and the biopsychosocial model to address each one of the risk and protective factors affecting GWG and its effect on birth outcomes. Include them in the search terms. Evaluate the relationship found between these factors using statistics | We share your concern that gestational weight gain could be affected by multiple factors including biological, psychological, behavioural, social and environmental factors. To address this, we have reviewed known factors associated with gestational weight gain, which have been addressed by studies conducted in sub-Saharan African countries. Studies conducted on gestational weight gain in sub-Saharan Africa mainly addressed behavioural factors rather than biological, psychological, social and environmental factors. The psychosocial variables such as depressive symptoms, perceived level of stress during pregnancy, self-efficacy, attitudes toward weight gain in pregnancy, feelings about motherhood, and career orientation were not addressed by any of the studies. The lack of socioecological and biopsychosocial variables prevented us from following the socioecological and the biopsychosocial model.  Variables such as pre-pregnancy weight, having antenatal care visits, engaging in physical activity, income, food consumption, knowledge about the importance of fruit, education, food supplementation, and seasonality have been addressed by different studies. However, these variables were measured differently across studies and we were unable to use statistics to evaluate the association between these factors and gestational weight gain. We have described the association using narrative synthesis. |
| 2. Consider cultural competency and variables specific for persons living in Sub-Saharan Africa, when evaluating articles regarding validity and reliability | While we agree with the comment, however, none of the studies included in this review reported on cultural competency or variables specific to people living in sub-Saharan. |
| 3. Evaluate the Obesity Paradox regarding GWG and birth outcomes. | Thank you for raising this important point. Studies showed that pre-pregnancy weight including obesity has been associated with gestational weight gain. We found no paradox regarding maternal obesity and gestational weight gain. Studies clearly reported that women with overweight or obesity are more likely to gain excess weight (S6 Fig). Studies that had higher number of women with overweight or obesity reported higher proportion of excessive gestational weight gain. However, most of the studies included in the review had no obese women or only a small number of obese women. Therefore, the finding of this study is not strong evidence to support or reject the Obesity Paradox hypothesis regarding gestational weight gain. |
| 4. Review statement: "recommendations on maternal weight management are inconsistent across countries..."   Note: Guidelines regarding appropriate levels of weight gain in pregnancy have been promoted worldwide. A variety of guidelines about the GWG exist. About half of the GWG guidelines are similar to the 2009 American Institutes of Medicine (IOM) and 73% of the EIRs are similar to the 2006 IOM. Reference: N, Haley S, Chow K, McDonald SD. Comparison of national gestational weight gain guidelines and energy intake recommendations. Obes Rev 2013; 14:68. | Thanks. We have revised as per your recommendation. |
| 5. Review statement: "To date, there are few systematic reviews and meta-analyses of research in sub-Saharan Africa (SSA) on the weight of pregnant women [29-31]. None addressed how much weight is gained during pregnancy by women in this population, or the effect on birth outcomes."   Note: Include in your search "self-monitor weight throughout pregnancy," "self-care," "person-centered care." | Thank you for your recommendation. We believe our search terms can trace any paper that addressed gestational weight gain in sub-Saharan Africa irrespective of the interventions (i.e., whether the intervention is self-monitored weight throughout pregnancy, self-care, person-centered care, or any other intervention such as food supplementations and physical exercise). We feel that specifically assessing self-monitoring of weight throughout pregnancy, self-care, person-centered care is beyond the scope of this review. |
| 6. Evaluate impact of Self-Care, Person-Centered Care, Person-Centered Medicine and People-Centered Public Health interventions on GWG and birth outcomes. How can these be implemented in Sub-Saharan Africa. | Thank you for pointing out this important topic. We found no study that addresses the impact of Self-Care, Person-Centered Care, Person-Centered Medicine and People-Centered Public Health interventions on gestational weight gain, matching our inclusion criteria. We also feel that evaluating the impact of Self-Care, Person-Centered Care, Person-Centered Medicine and People-Centered Public Health interventions on birth outcomes and assessing the implementation method is beyond the scope of this review. We will consider doing this in the future as a separate paper. |

| **Reviewer 2 comments** | **Authors' reply** |
| --- | --- |
| I reviewed the manuscript “Gestational Weight Gain and its Effect on Birth Outcomes in sub-Saharan Africa: Systematic Review and Meta-analysis”. The author(s) of this paper sought to present a review that determines the distribution of gestational weight gain (GWG) across African countries and its association with birth outcomes. Overall, I think this paper is well-written, and merits consideration for publication.  Some of the papers main strengths include a very clear presentation of findings of the studies considered in the review as well as meticulously critiquing each of the papers, identifying their merits and demerits. I, however, have some minor comments about issues the authors can clarify or address. Some of my concerns are outlined as below: | We thank you for committing your time to read through the manuscript; pointing out the strengths of the manuscript; and providing your comments for further improvements. We have addressed your concerns under each point. |
| Minor comments: |  |
| Study selection procedure  I strongly suggest that the authors remove the names from the body of the article. Stating that “the authors screened the studies …” should suffice. The authors have clearly spelled out how they each contributed to the article at the end of it.  Additionally, I don’t really think disagreements between authors and how they were resolved have any essence in the article. Please remove it. | We have accepted your comment and removed the authors’ name from the body of the article. |
| Data extraction process  Again, remove the names of specific authors. I think all the authors bear responsibility for the merits and the demerits of the methods and procedures used in this study. | Thank you. We have removed the names of specific authors. |
| Study characteristics  I suggest the authors start a new sentence after Congo before proceeding to present the categories of the countries based  On the income status. As it looks now, it reads as if the countries categorized based on income status are in addition to  Those listed by names. I would suggest starting the new sentence with “Based on the income status, these countries can  be grouped into …” | Thank you. We have accepted the comment and corrected it. |
| Effect of GWG on birth outcomes  Low birthweight  While the results of Nenomsa et al. [39] show quite clear relationship between GWG and birth weight, Fouelifack et al. [41] seem to present quite a different picture. Were there any reasons presented in the study that could have accounted  for a greater proportion of women who gained adequate GWG giving birth to LBW babies than those who did not gain adequate GWG? | Thank you. We agree with the reviewer. These two studies presented quite different results. Fouelifack et al. study not discuss reasons that could have contributed to their findings. However, there are some differences regarding study participant characteristics in Nenomsa et al and Fouelifack et al. In Nenomsa et al, about 17% were either overweight or obese, while 47% were either overweight or obese in Fouelifack et al. In addition, only 2.7% gained excessive weight in Nenomsa et al, while 32% gained excessive weight in Fouelifack et al. Both studies were conducted in different settings. All these factors could have contributed to their differences. |
| Discussion  On page 23, change this sentence in the second paragraph:  “Firstly, the measurement of pre-pregnancy weight of the women was problematic, with four studies [9, 41, 52, 54] using self-reported pre-pregnancy weight. However, because there is a typically a difference between self-reported weight and actual measured weight [82-84].”  “Women tend to under-report their pre pregnancy weight [85, 86], and…”  Substantively, I would suggest caution with attributing studies [85] and [86] to a study in sub-Saharan Africa. This is because I believe there is a social and cultural component that may render this statement not necessarily true for African women (there is quite a lot I could try to explain here but I will let you do that work on your end). Weight gain is not necessarily perceived as negative as it is in countries like the USA. I, therefore, suspect that women will be less likely inclined to deliberate understate their weight. | Thanks for your comment. We agree and have deleted the sentence (Women tend to under-report their pre pregnancy weight [85, 86]) including reference 85 and 86. |
| Summing it all up  I enjoyed reading the paper. It critiques that other studies very well, while it also does a significant amount of work outlining its own limitations. I suggest you consider the comments above and it will make an interesting read for your audience. | Thank you so much for your comments. |

| **Reviewer 3 comments** | **Authors' reply** |
| --- | --- |
| This is an interesting review. Please find my comments on how to improve it | Thank you for your comments. |
| 1. ABSTRACT: better clarify why only 2 studies were included in the meta-analysis | We included 16 studies in the meta analysis to pool proportions of inadequate, adequate and excess gestational weight gain (S1-3 Figs). However, two studies were used to evaluate the association of gestational weight gain with pre-eclampsia and macrosomia. Because, these two studies were the only studies that reported the number of women who experienced pre-eclampsia and gave birth to macrosomic babies among women who gained inadequate, adequate and excess gestational weight. We have clarified this in the abstract |
| 2. Inclusion criteria. Add information on whether language was or not an exclusion criteria | We have included the recommended information. |
| 3. Quality assessment of studies: I suggest to add details on the tool used | We have added more details on the tools used. |
| 4. Data analysis. Please better clarify this sentence. Providing few examples “For studies that used arbitrary classifications, we used the authors’ own classifications. | We have provided examples and clarified it. |
| 5. Terminology: I fell that “inadequate” GWG is confusing, and I suggest to modify this in “insufficient and “excessive” | While we agree with your concern, we used the Institute of Medicine recommendation to determine the adequacy or sufficiency of gestational weight gain. To avoid confusion, we preferred to use the terms inadequate, adequate and excess gestational weight gain to make it consistent with the Institute of Medicine terminologies and most other studies on gestational weight gain. |
| 6. Critical appraisal results: you can delete the following sentence which is already stated in methods “We used the Effective Public Health Practice Project Quality Assessment Tool for Quantitative Studies to assess the quality of the included studies | We have deleted it. |
| 7. In methods will we good to further detail how you did analysed factors associated with weight gain | We were unable to analyse the association between gestational weight gain and different factors because these factors were classified differently among different studies. We used narrative synthesis to describe these factors. We included this information in the method section. |
| 8. Discussion: I suggest to shorten it down | Thank you for your suggestion. As much as possible we have tried to limit the information which we need to discuss or put in the discussion section. |
| 9. Discussion: will be good to add a point in relation on how are the WHO recommendation on weight gain applicable (ie, based on evidence) to the African population | Thank you for your suggestion. We have conducted a search for gestational weight gain recommendations by WHO, but found no document. We would be interested to learn if there are gestational weight gain recommendations by WHO for African populations or any other specific populations. If the reviewer is aware of any gestational weight gain recommendation by WHO, we are happy to include this point in our discussion. |
| 10. Discussion: add recommendations for policy makers and for researchers | We have recommended researchers to conduct methodologically rigorous studies in sub-Saharan Africa to answer GWG related research questions including what factors affect GWG and the association between GWG and birth outcomes (page 30, at the end of first paragraph). In addition, we suggested policy makers to facilitate the development of GWG guidelines to avoid arbitrary classifications of GWG. |
