# Supplementary material for: Gestational weight gain and its effect on birth outcomes in sub-Saharan Africa: Systematic review and meta-analysis
Source: PLoS One. 2020 Apr 23;15(4):e0231889. doi: 10.1371/journal.pone.0231889 (PMC7179909; doi:10.1371/journal.pone.0231889)
Supplement: Attachment — Submitted filename: Response to Reviewers.docx [file pone.0231889.s013.docx]

Title:	Gestational Weight Gain and its Effect on Birth Outcomes in sub-Saharan Africa: Systematic Review and Meta-analysis

Authors

1. Fekede Asefa Kumsa: [sinboona@gmail.com](mailto:sinboona@gmail.com)
2. Allison Cummins: [Allison.cummins@uts.edu.au](mailto:Allison.cummins@uts.edu.au)
3. Yadeta Dessie: [yad_de2005@yahoo.com](mailto:yad_de2005@yahoo.com)
4. Andrew Hayen: [Andrew.hayen@uts.edu.au](mailto:Andrew.hayen@uts.edu.au)
5. Maralyn Foureur: [Maralyn.Foureur@health.nsw.gov.au](mailto:Maralyn.Foureur@health.nsw.gov.au)

Dear editor,

We are very grateful to the editor and the reviewers who reviewed the manuscript. We have revised the manuscript as per the reviewers’ comment and described amendments made on the manuscript in the following table.

Sincerely

| **Reviewer 1 comments** | **Authors' reply** |
| --- | --- |
| Reviewer #1: Very interesting and well written article. Please review the following terms: "macrosomic baby" and consider writing instead "a baby born with macrosomia." Let us remember that the medical condition does not define the person. Thank you | Thank you. We accepted your recommendation and changed the terms: "macrosomic baby" to "a baby born with macrosomia." |
